# Supplementary material for: A Cysteine Residue within the Kinase Domain of Zap70 Regulates Lck Activity and Proximal TCR Signaling
Source: Cells. 2022 Sep 1;11(17):2723. doi: 10.3390/cells11172723 (PMC9455082; doi:10.3390/cells11172723)
Supplement: Supplementary file 1 [file cells-11-02723-s001.zip › cells-1863427-supplementary.pdf]

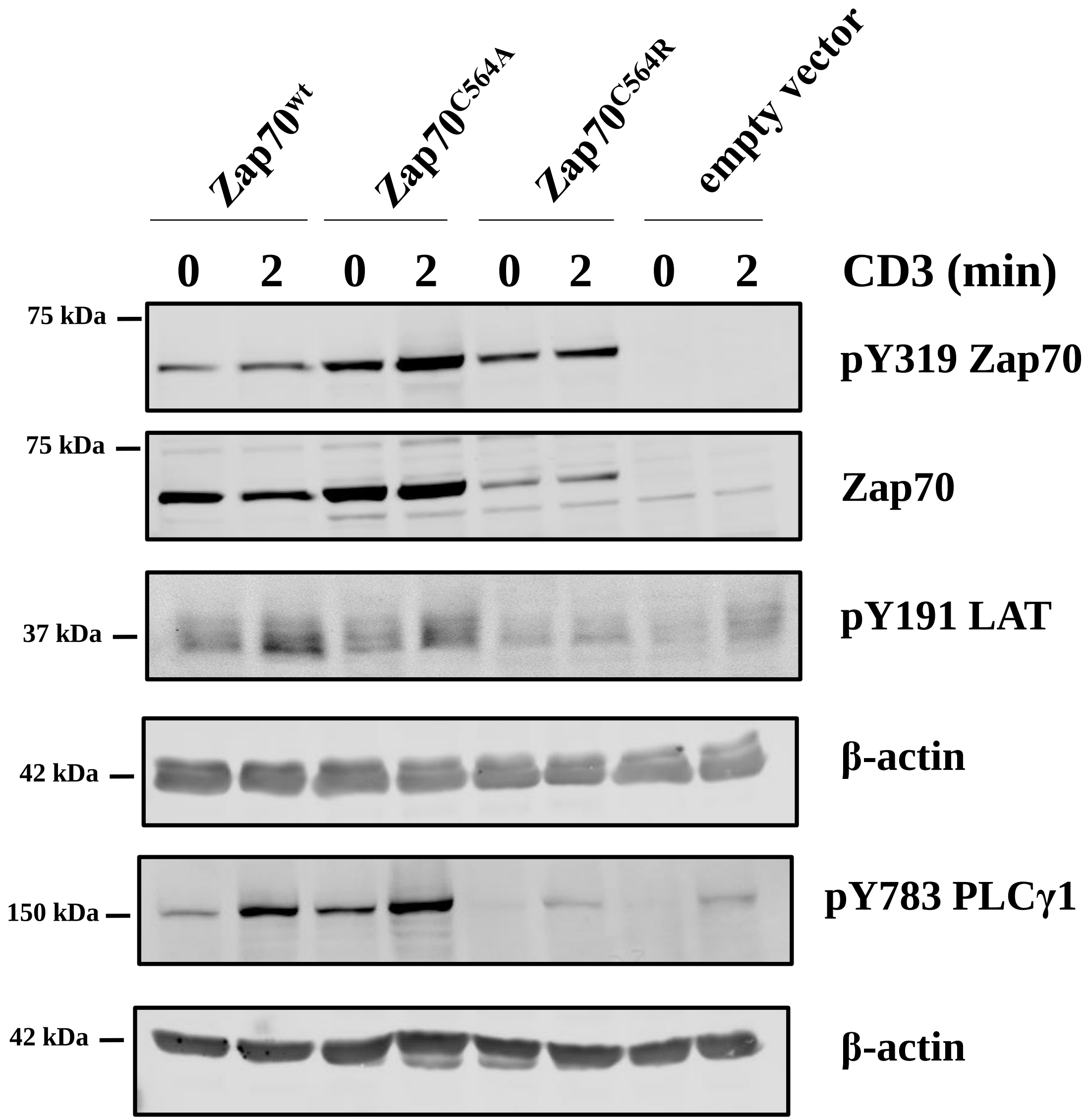

Figure S1. Analysis of TCR signaling in cells expressing C564A and C564R Zap70 mutants.
P116 cells were transiently transfected with the indicated plasmids. Subsequently, cells were stimulated with UCHT1 for 2 min, lysed and the phosphorylation of the indicated molecules was analyzed using phospho-specific antibodies. Equal protein loading was assessed using antibodies directed against β-actin. One representative experiment is shown (n=3).

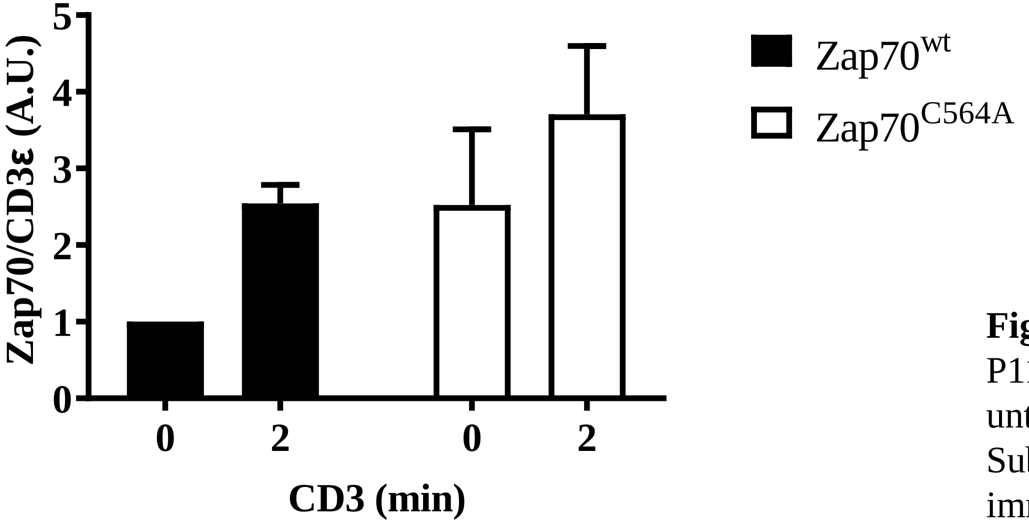

Figure S2. Recruitment of Zap70 to the ζ-chain.
P116 cells stably expressing Zap70^{wt} or Zap70^{C564A} were either left untreated or stimulated with an anti-CD3 antibody (UCHT1). Subsequently, cells were lysed and the ζ-chain was immunoprecipitated. Zap70 immunoblots were performed to evaluate the association between Zap70 and the TCR. The efficiency of the immunoprecipitation was evaluated by performing anti-CD3ε immunoblots. The Zap70 signal was normalized to that of CD3ε. Values were further normalized by setting to one the Zap70/CD3ε ratio form unstimulated cells expressing Zap70^{wt}. The graph shows analyses of the association between Zap70 and the ζ-chain expressed as arbitrary units ± SEM of four independent experiments. Statistical analyses performed using the Welchs *t* test revealed no significant difference between unstimulated cells expressing Zap70^{wt} or Zap70^{C564A} and also no significant difference between CD3-stimulated cells expressing Zap70^{wt} or Zap70^{C564A}.

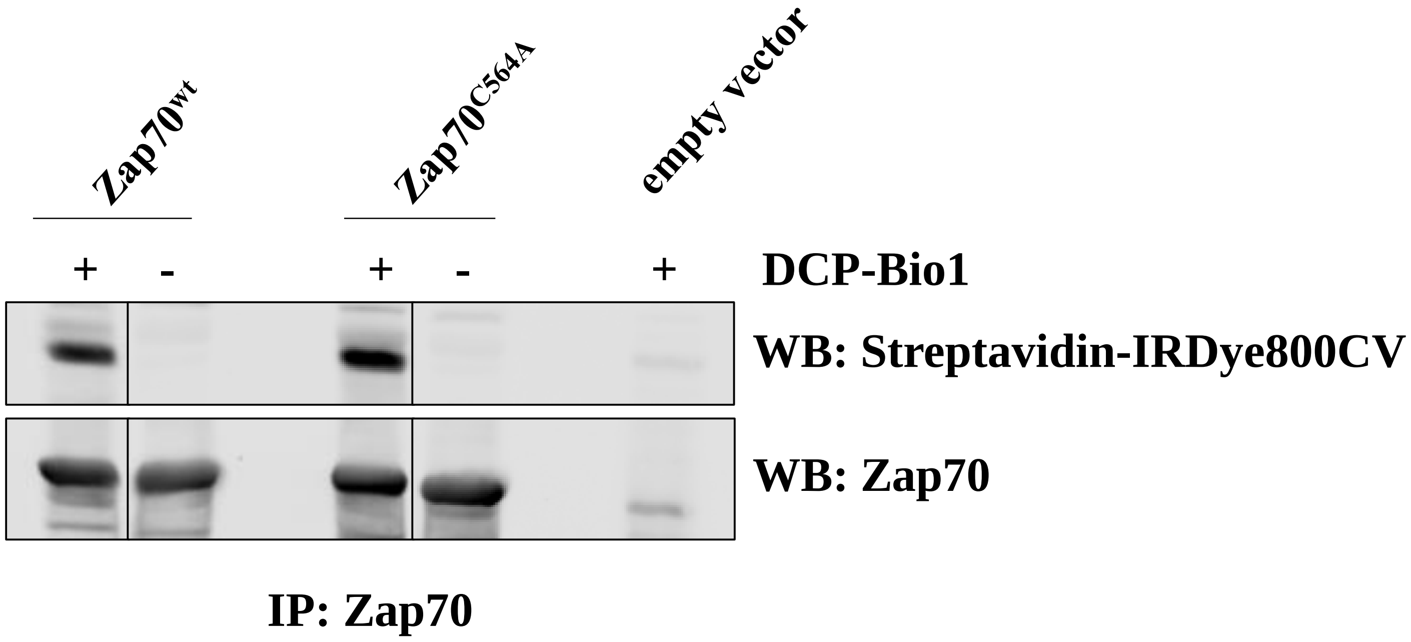

Figure S3. Analysis of Zap70 sulfenylation.
P116 cells stably expressing Zap70^{wt}, Zap70^{C564A}, or no Zap70 (empty vector) were either left untreated or incubated with DCP-Bio1 as previously described [16]. Subsequently, cells were lysed and Zap70 was immunoprecipitated. The sulfenylation levels were detected using Streptavidin-IRDye800CV. Zap70 immunoblot was performed to show equal loading. One representative experiment is shown (n=3).
